# 5-(4-Bromo-2-nitro­phen­yl)-1,3,4-thia­diazol-2-amine

**DOI:** 10.1107/S1600536811030868

**Published:** 2011-08-06

**Authors:** Jian-qiang Zhang, Qiu He, Qianghua Jiang, Haipin Mu, Rong Wan

**Affiliations:** a Department of Applied Chemistry, College of Science, Nanjing University of Technology, No. 5 Xinmofan Road, Nanjing, Nanjing 210009, People’s Republic of China

## Abstract

The title compound, C_8_H_5_BrN_4_O_2_S, was synthesized by the reaction of 4-bromo-2-nitro­benzoic acid with thio­semi­carbazide. The dihedral angle between the thia­diazole and benzene rings is 40.5 (2)°. In the crystal, the strongest N—H⋯N inter­molecular hydrogen bond, between the amine group and one thia­diazole N atom, forms centrosymmetric dimers. The other amine H atom extends the supra­molecular network, forming an N—H⋯N contact with the other thia­diazole N atom.

## Related literature

For the biological activity of 1,3,4-thia­diazole derivatives, see: Nakagawa *et al.* (1996 ▶); Wang *et al.* (1999 ▶).
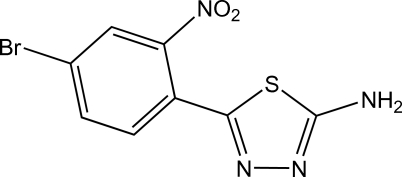


## Experimental

### 

#### Crystal data

C_8_H_5_BrN_4_O_2_S
*M* *_r_* = 301.13
Monoclinic, 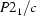

*a* = 11.231 (2) Å
*b* = 9.2580 (19) Å
*c* = 10.868 (2) Å
β = 113.08 (3)°
*V* = 1039.6 (4) Å^3^
*Z* = 4
Mo *K*α radiation
μ = 4.14 mm^−1^
*T* = 293 K
0.20 × 0.10 × 0.10 mm

#### Data collection

Enraf–Nonius CAD-4 diffractometer
Absorption correction: ψ scan (North *et al.*, 1968 ▶) *T*_min_ = 0.491, *T*_max_ = 0.682
3909 measured reflections
1920 independent reflections
1409 reflections with *I* > 2σ(*I*)
*R*_int_ = 0.116
3 standard reflections every 200 reflections  intensity decay: 1%

#### Refinement

*R*[*F* ^2^ > 2σ(*F* ^2^)] = 0.049
*wR*(*F* ^2^) = 0.104
*S* = 1.01
1920 reflections
152 parameters
H atoms treated by a mixture of independent and constrained refinement
Δρ_max_ = 0.55 e Å^−3^
Δρ_min_ = −0.97 e Å^−3^

### 

Data collection: *CAD-4 EXPRESS* (Enraf–Nonius, 1989 ▶); cell refinement: *CAD-4 EXPRESS*; data reduction: *XCAD4* (Harms & Wocadlo,1995 ▶); program(s) used to solve structure: *SHELXS97* (Sheldrick, 2008 ▶); program(s) used to refine structure: *SHELXL97* (Sheldrick, 2008 ▶); molecular graphics: *SHELXTL* (Sheldrick, 2008 ▶); software used to prepare material for publication: *SHELXL97*.

## Supplementary Material

Crystal structure: contains datablock(s) global, I. DOI: 10.1107/S1600536811030868/bh2371sup1.cif

Structure factors: contains datablock(s) I. DOI: 10.1107/S1600536811030868/bh2371Isup2.hkl

Supplementary material file. DOI: 10.1107/S1600536811030868/bh2371Isup3.cml

Additional supplementary materials:  crystallographic information; 3D view; checkCIF report

## Figures and Tables

**Table 1:** Hydrogen-bond geometry (Å, °)

*D*—H⋯*A*	*D*—H	H⋯*A*	*D*⋯*A*	*D*—H⋯*A*
N4—H4*B*⋯N3^i^	0.79 (7)	2.25 (7)	3.014 (6)	165 (7)
N4—H4*C*⋯N2^ii^	0.80 (6)	2.34 (6)	3.103 (6)	161 (6)

Symmetry codes: (i) 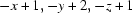; (ii) 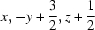.

**Fig. 1.**
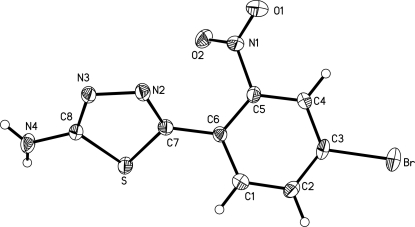
A view of the molecular structure of the title compound, with displacement ellipsoids drawn at the 50% probability level.

**Fig. 2.**
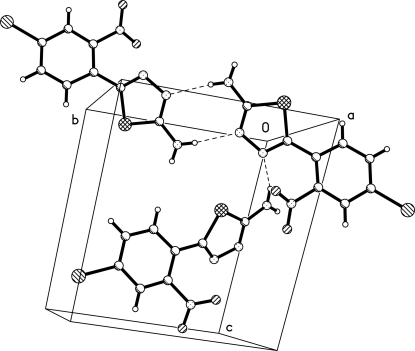
Partial packing view showing the hydrogen bonds network. Dashed lines indicate intermolecular N—H···N hydrogen bonds.

## supplementary crystallographic information

### Comment

1,3,4-Thiadiazole derivatives represent an interesting class of compounds possessing a broad spectrum of biological activities (Nakagawa *et al.*, 1996). These compounds are known to exhibit diverse biological activities, such as insecticidal and fungicidal activities (Wang *et al.*, 1999). Here we report the crystal structure of the title compound, a new thiadiazole. In the molecular structure (Fig. 1) the bond lengths and angles are within normal ranges. Thiadiazole ring C8/S/C7/N2/N3 is planar, and the mean deviation from the plane is 0.0046 Å. The dihedral angle between the thiadiazole and benzene rings is 40.5 (2)°. In the crystal structure, the strongest N—H···N intermolecular contact (first entry in the hydrogen bonds Table) forms centrosymmetric dimers in the crystal (top molecules in Fig. 2). This pattern is the primary supramolecular structure for this compound. The other hydrogen bond (entry 2) is comparatively weak, and extends the primary pattern to a three-dimensional network, which may be effective in the stabilization of the crystal structure.

### Experimental

4-Bromo-2-nitrobenzoic acid (2 mmol) and thiosemicarbazide (5 mmol) were mixed in a 25 ml flask, and kept in the oil bath at 90 °C for 6 h. After cooling, the crude product precipitated and was filtered. Pure compound was obtained by crystallization from ethanol (20 ml). Single crystals suitable for X-ray diffraction were obtained by slow evaporation of an acetone solution.

### Refinement

All H atoms bonded to the C atoms were placed geometrically at the distances of 0.93 Å and included in the refinement in riding motion approximation with *U*_iso_(H) = 1.2*U*_eq_(carrier C atom). Amine H atoms H4B and H4C were found in a difference map and refined freely, with *U*_iso_(H) = 1.2*U*_eq_(N4).

### Crystal data

**Table:**

C_8_H_5_BrN_4_O_2_S	*F*(000) = 592
*M**_r_* = 301.13	*D*_x_ = 1.924 Mg m^−^^3^
Monoclinic, *P*2_1_/*c*	Melting point: 506 K
Hall symbol: -P 2ybc	Mo *K*α radiation, λ = 0.71073 Å
*a* = 11.231 (2) Å	Cell parameters from 25 reflections
*b* = 9.2580 (19) Å	θ = 10–13°
*c* = 10.868 (2) Å	µ = 4.14 mm^−^^1^
β = 113.08 (3)°	*T* = 293 K
*V* = 1039.6 (4) Å^3^	Block, yellow
*Z* = 4	0.20 × 0.10 × 0.10 mm

### Data collection

**Table:**

Enraf–Nonius CAD-4 diffractometer	1409 reflections with *I* > 2σ(*I*)
Radiation source: fine-focus sealed tube	*R*_int_ = 0.116
graphite	θ_max_ = 25.4°, θ_min_ = 2.0°
ω/2θ scans	*h* = −13→12
Absorption correction: ψ scan (North *et al.*, 1968)	*k* = −11→11
*T*_min_ = 0.491, *T*_max_ = 0.682	*l* = 0→13
3909 measured reflections	3 standard reflections every 200 reflections
1920 independent reflections	intensity decay: 1%

### Refinement

**Table:**

Refinement on *F*^2^	Secondary atom site location: difference Fourier map
Least-squares matrix: full	Hydrogen site location: inferred from neighbouring sites
*R*[*F*^2^ > 2σ(*F*^2^)] = 0.049	H atoms treated by a mixture of independent and constrained refinement
*wR*(*F*^2^) = 0.104	*w* = 1/[σ^2^(*F*_o_^2^) + (0.025*P*)^2^] where *P* = (*F*_o_^2^ + 2*F*_c_^2^)/3
*S* = 1.01	(Δ/σ)_max_ < 0.001
1920 reflections	Δρ_max_ = 0.55 e Å^−^^3^
152 parameters	Δρ_min_ = −0.97 e Å^−^^3^
0 restraints	Extinction correction: *SHELXL97* (Sheldrick, 2008), Fc^*^=kFc[1+0.001xFc^2^λ^3^/sin(2θ)]^-1/4^
0 constraints	Extinction coefficient: 0.0114 (8)
Primary atom site location: structure-invariant direct methods	

### Fractional atomic coordinates and isotropic or equivalent isotropic displacement parameters (Å^2^)

**Table:**

	*x*	*y*	*z*	*U*_iso_*/*U*_eq_	
Br	0.11486 (5)	0.01063 (5)	0.19612 (6)	0.0512 (2)	
S	0.34305 (13)	0.66328 (12)	0.58298 (11)	0.0389 (3)	
C1	0.2933 (5)	0.3456 (5)	0.4493 (4)	0.0398 (11)	
H1A	0.3437	0.3551	0.5402	0.048*	
N1	0.1367 (4)	0.5710 (4)	0.1441 (4)	0.0370 (9)	
O1	0.1374 (4)	0.5588 (4)	0.0330 (3)	0.0585 (10)	
O2	0.0937 (3)	0.6740 (3)	0.1818 (3)	0.0517 (10)	
N2	0.3728 (4)	0.6948 (4)	0.3620 (3)	0.0357 (9)	
C2	0.2492 (5)	0.2112 (5)	0.3991 (5)	0.0418 (12)	
H2B	0.2717	0.1308	0.4549	0.050*	
N3	0.4262 (4)	0.8176 (4)	0.4373 (3)	0.0339 (9)	
C3	0.1724 (4)	0.1958 (4)	0.2670 (5)	0.0360 (10)	
C4	0.1413 (5)	0.3137 (5)	0.1816 (4)	0.0339 (11)	
H4A	0.0920	0.3029	0.0907	0.041*	
N4	0.4674 (5)	0.9189 (5)	0.6472 (4)	0.0460 (12)	
H4B	0.486 (6)	0.996 (7)	0.630 (6)	0.055*	
H4C	0.446 (5)	0.910 (6)	0.709 (6)	0.055*	
C5	0.1853 (4)	0.4459 (5)	0.2350 (4)	0.0331 (10)	
C6	0.2654 (4)	0.4671 (4)	0.3696 (4)	0.0283 (9)	
C7	0.3245 (4)	0.6062 (5)	0.4235 (4)	0.0303 (9)	
C8	0.4186 (4)	0.8148 (4)	0.5534 (4)	0.0318 (10)	

### Atomic displacement parameters (Å^2^)

**Table:**

	*U*^11^	*U*^22^	*U*^33^	*U*^12^	*U*^13^	*U*^23^
Br	0.0612 (3)	0.0292 (3)	0.0646 (4)	−0.0078 (3)	0.0262 (3)	−0.0142 (2)
S	0.0593 (7)	0.0333 (6)	0.0300 (5)	−0.0143 (6)	0.0240 (5)	−0.0042 (5)
C1	0.045 (3)	0.037 (2)	0.031 (2)	0.001 (2)	0.008 (2)	0.000 (2)
N1	0.037 (2)	0.036 (2)	0.031 (2)	0.001 (2)	0.0059 (17)	0.0040 (17)
O1	0.073 (2)	0.056 (2)	0.0359 (18)	−0.001 (2)	0.0100 (17)	0.0107 (18)
O2	0.060 (2)	0.0277 (16)	0.057 (2)	0.0082 (18)	0.0115 (19)	0.0035 (16)
N2	0.047 (2)	0.0326 (17)	0.0289 (18)	−0.010 (2)	0.0163 (17)	−0.0061 (16)
C2	0.046 (3)	0.026 (2)	0.044 (3)	−0.003 (2)	0.008 (2)	0.005 (2)
N3	0.045 (2)	0.0318 (18)	0.0267 (18)	−0.0118 (19)	0.0163 (17)	−0.0039 (15)
C3	0.034 (2)	0.0213 (18)	0.053 (3)	−0.005 (2)	0.017 (2)	−0.009 (2)
C4	0.039 (3)	0.031 (2)	0.028 (2)	−0.002 (2)	0.0086 (19)	−0.0053 (18)
N4	0.076 (3)	0.036 (2)	0.033 (2)	−0.023 (2)	0.028 (2)	−0.0124 (19)
C5	0.034 (2)	0.0246 (18)	0.035 (2)	−0.001 (2)	0.007 (2)	−0.0030 (19)
C6	0.034 (2)	0.0274 (19)	0.0261 (19)	−0.005 (2)	0.0140 (17)	−0.0026 (17)
C7	0.034 (2)	0.031 (2)	0.028 (2)	−0.004 (2)	0.0140 (19)	−0.0012 (18)
C8	0.035 (2)	0.027 (2)	0.031 (2)	−0.007 (2)	0.0109 (19)	−0.0013 (18)

### Geometric parameters (Å, °)

**Table:**

Br—C3	1.887 (4)	C2—C3	1.362 (7)
S—C8	1.734 (4)	C2—H2B	0.9300
S—C7	1.744 (4)	N3—C8	1.297 (5)
C1—C2	1.371 (6)	C3—C4	1.386 (6)
C1—C6	1.379 (6)	C4—C5	1.361 (6)
C1—H1A	0.9300	C4—H4A	0.9300
N1—O2	1.210 (5)	N4—C8	1.353 (6)
N1—O1	1.215 (5)	N4—H4B	0.79 (6)
N1—C5	1.481 (6)	N4—H4C	0.81 (6)
N2—C7	1.303 (5)	C5—C6	1.399 (6)
N2—N3	1.392 (4)	C6—C7	1.462 (6)
			
C8—S—C7	86.4 (2)	C3—C4—H4A	121.0
C2—C1—C6	122.2 (4)	C8—N4—H4B	122 (4)
C2—C1—H1A	118.9	C8—N4—H4C	113 (4)
C6—C1—H1A	118.9	H4B—N4—H4C	119 (6)
O2—N1—O1	124.7 (4)	C4—C5—C6	123.3 (4)
O2—N1—C5	118.8 (4)	C4—C5—N1	116.2 (3)
O1—N1—C5	116.4 (4)	C6—C5—N1	120.4 (4)
C7—N2—N3	112.4 (3)	C1—C6—C5	115.9 (4)
C3—C2—C1	119.7 (4)	C1—C6—C7	120.7 (3)
C3—C2—H2B	120.2	C5—C6—C7	123.2 (4)
C1—C2—H2B	120.2	N2—C7—C6	124.3 (4)
C8—N3—N2	112.3 (3)	N2—C7—S	114.1 (3)
C2—C3—C4	120.8 (4)	C6—C7—S	121.6 (3)
C2—C3—Br	119.9 (3)	N3—C8—N4	123.8 (4)
C4—C3—Br	119.1 (3)	N3—C8—S	114.8 (3)
C5—C4—C3	118.0 (4)	N4—C8—S	121.3 (3)
C5—C4—H4A	121.0		
			
C6—C1—C2—C3	−1.8 (8)	N1—C5—C6—C1	172.8 (4)
C7—N2—N3—C8	1.5 (5)	C4—C5—C6—C7	172.7 (5)
C1—C2—C3—C4	2.1 (8)	N1—C5—C6—C7	−11.3 (7)
C1—C2—C3—Br	178.4 (4)	N3—N2—C7—C6	−178.4 (4)
C2—C3—C4—C5	−2.8 (7)	N3—N2—C7—S	−1.3 (5)
Br—C3—C4—C5	−179.2 (4)	C1—C6—C7—N2	135.9 (5)
C3—C4—C5—C6	3.5 (8)	C5—C6—C7—N2	−39.8 (7)
C3—C4—C5—N1	−172.6 (4)	C1—C6—C7—S	−40.9 (6)
O2—N1—C5—C4	130.8 (5)	C5—C6—C7—S	143.4 (4)
O1—N1—C5—C4	−45.7 (6)	C8—S—C7—N2	0.7 (4)
O2—N1—C5—C6	−45.5 (6)	C8—S—C7—C6	177.8 (4)
O1—N1—C5—C6	138.1 (5)	N2—N3—C8—N4	177.6 (4)
C2—C1—C6—C5	2.3 (7)	N2—N3—C8—S	−1.0 (5)
C2—C1—C6—C7	−173.7 (5)	C7—S—C8—N3	0.2 (4)
C4—C5—C6—C1	−3.1 (7)	C7—S—C8—N4	−178.4 (4)

### Hydrogen-bond geometry (Å, °)

**Table:**

*D*—H···*A*	*D*—H	H···*A*	*D*···*A*	*D*—H···*A*
N4—H4B···N3^i^	0.79 (7)	2.25 (7)	3.014 (6)	165 (7)
N4—H4C···N2^ii^	0.80 (6)	2.34 (6)	3.103 (6)	161 (6)

Symmetry codes: (i) −*x*+1, −*y*+2, −*z*+1; (ii) *x*, −*y*+3/2, *z*+1/2.
